# Tissue mechanics and expression of TROP2 in oral squamous cell carcinoma with varying differentiation

**DOI:** 10.1186/s12885-020-07257-7

**Published:** 2020-08-27

**Authors:** Baoping Zhang, Shuting Gao, Ruiping Li, Yiting Li, Rui Cao, Jingyang Cheng, Yumeng Guo, Errui Wang, Ying Huang, Kailiang Zhang

**Affiliations:** 1 Department (Hospital) of Stomatology, Lanzhou University, Donggang west Road 199, Lanzhou, 730000 Gansu China; 2 Institute of Biomechanics and Medical Engineering, Lanzhou University, Lanzhou, 730000 China

**Keywords:** Oral squamous cell carcinoma, TROP2, Tissue stiffness, Differentiation, Survival

## Abstract

**Background:**

Trophoblast cell surface antigen 2 (TROP2) is overexpressed in many squamous cell carcinomas and promotes tumor development and invasion. The association between TROP2 expression and occurrence and development of oral squamous cell carcinoma (OSCC) remains to be understood.

**Methods:**

We investigated the role of TROP2 in OSCC patients using a combination of biophysical approaches. A total of 108 OSCC patient specimens with varying degrees of differentiation were subjected to hematoxylin and eosin staining, immunohistochemistry, Kaplan-Meier survival curve analysis, and atomic force microscopy to analyze TROP2 expression, morphology, and mechanical properties of OSCC tissues.

**Results:**

TROP2 was overexpressed in 34% of poorly differentiated OSCC samples. High levels of TROP2 were associated with 10.2% survival rate lower than 45.4% and patient age (odds ratio [OR] = 0.437, *P* = 0.039, 95% confidence interval [CI, 0.198–0.966]), tumor size (OR = 13.148, *P* = 0.000, 95% CI [5.060–34.168]), and TNM stage (OR = 0.141, *P* = 0.000, 95% CI [0.082–0.244]). Average surface roughness of low, medium, and highly differentiated OSCC tissues were 448.9 ± 54.8, 792.7 ± 83.6, and 993.0 ± 104.3 nm, respectively. The Pearson coefficient revealed a negative association between tumor stiffness and TROP2 expression (*r* = − 0.84, *P* < 0.01).

**Conclusion:**

Overexpression of TROP2 negatively associated with patient survival, degree of tumor differentiation, and tissue mechanics. Taken together, our findings demonstrated that TROP2 may be an indicator of OSCC differentiation leading to the altered mechanical properties of OSCC tissues.

## Background

Oral squamous cell carcinoma (OSCC) is a common subtype of head and neck and other malignant tumors [1, 2]. The past few decades have shown increased incidence of OSCC that is expected to rise further in the future [3]. Therefore, it is imperative to determine biological factors associated with the early diagnosis and treatment of OSCC.

Human trophoblast cell surface antigen 2 (TROP2), also called tumor-associated calcium signal transduction-2 (TACSTD-2), is a surface glycoprotein encoded by TACSTD that has extracellular domains, a single transmembrane domain, and a short tail [4, 5]. TROP2 is overexpressed in many human cancers, including ovarian [6, 7], gastric [8, 9], colorectal [10], pancreatic [11], and laryngeal cancers [12]. Inhibiting TROP2 expression has shown promise in clinical applications [13, 14]. TROP2 regulates tumorigenic properties including cancer cell adhesion, invasion, and migration. Tang et al. [15] have recently shown that TROP2 impacts growth and metastasis by activating PI3K/AKT signaling. This phenomenon has also been observed in gallbladder cancer [16]. Among the various biochemical mechanisms involved in tumorigenesis, the role of β-catenin has been studied extensively [8, 17–19]. This has shed light on the biological functions of TROP2 and its use as a prognostic biomarker for OSCC.

Atomic force microscopy (AFM) is a powerful tool that generates surface topographical images with magnifications that range between macro- and nanoscales. AFM has been used to determine the mechanical properties of tumor tissues in a variety of cancers, such as those of the breast [20], liver [21], and lung [22]. Parameters for tissue stress, such as mechanical phenotype index, correlate with cancer development and invasion [23]. Advancements in technology used for determining biophysical properties have facilitated the nano-level analysis of tumor tissues.

This study aims at investigating the correlation between TROP2 expression and clinicopathological characteristics of OSCC. We have demonstrated the tissue morphology and mechanics of OSCC samples during tumor development using AFM. We believe our findings will help develop TROP2 in accurately diagnosing OSCC in tumors with different grades of differentiation.

## Methods

### Tissue preparation

The protocols in this study were approved by the research ethics committee of Lanzhou University. Tumor samples were collected from patients after obtaining written informed consent. A total of 108 patients with oral squamous cell carcinoma (OSCC) were registered at the second hospital of Lanzhou University between January 2013 and March 2019. Among these samples, 36 samples each showed high, moderate, and low levels of differentiation. The experimental group comprised 60 males and 48 females aged 41–68 years (average: 51 years). All patients were diagnosed with OSCC based on surgery and pathology; patients did not undergo radiotherapy, chemotherapy, or immunotherapy before surgery. Pathological analysis after tumor biopsy was performed by two experienced pathologists, after which the diagnosis of other diseases (including inflammation at other sites and secondary tumors) were excluded. Cancer and cervical lymph node tissues were collected after maxillofacial surgery. All specimens were sampled from typical areas of the lesion and fixed with 10% neutral-buffered formalin followed by conventional paraffin embedding. Among them, 42 and 66 patients exhibited no and cervical lymph node metastases, respectively. Clinical TNM staging was performed according to the 7th edition TNM staging classification standard jointly developed by the International Union for Cancer Control and American Joint Committee on Cancer [24] and World Health Organization guidelines [25].

### Hematoxylin and eosin (H&E) staining

OSCC tissues were fixed overnight using 10% neutral formalin (Solarbio, Beijing, China), paraffin embedded, sliced into 4-μm thick sections, dewaxed using xylene, and rehydrated using different concentrations of ethanol. The sections were stained with hematoxylin for 5 min and hydrochloric acid-ethanol and eosin for 3 min followed by gradient dehydration, transparentization, sealing, and neutral resin sealing (Solarbio, Beijing, China). Sections were visualized and imaged using the Olympus BX53 at magnifications of × 10, 20, and 40.

### Immunohistochemistry

HE sections were subjected to the S-P method to detect TROP2 expression. The sections were incubated overnight with the primary antibody against TROP2 (1:1000, Abcam, USA) at 4 °C followed by incubation with biotin-labeled goat anti-rabbit IgG (1:5000, Abcam, USA) at 37 °C for 1 h. The sections were then developed using DAB (Beijing Zhongshan Golden Bridge Biotechnology, China), dehydrated, transparentization, and film and neutral resin sealed. The prepared sections were visualized using microscopy (Olympus BX53, Japan).

### AFM

Fixed tissues were placed in Petri dishes containing phosphate-buffered saline. All analyses for mechanical properties were performed using the biological atomic force microscope (BioAFM; NanoWizard III, Bruker, USA). Silicon AFM probes from the Pointprobe® series with a force constant of 0.2 N/m (CONTR-reflex coating, NanoWorld, USA) were used. The spring constant of the probe was calibrated using built-in thermal vibration before measuring the resonance frequency of 13 kHz and thickness of 2 μm. AFM was performed using the contact model and a scan rate of 0.5 Hz/s in air. Force-distance curves are generated when the probe contacts the tissue following which, the structure, morphology, and mechanical properties of samples are measured at 5 μm/s [26]. Six random sites were selected for each sample and each site was measured 15 times. We used the modified Hertz contact model to analyze force-distance curves [27] and calculate Young’s modulus and roughness of OSCC tissues with varying differentiation.

### Statistical analysis

Statistical analyses were performed using SPSS 22.0 (Statistical Product and Service Solutions, IBM). Force spectrum data were expressed as mean ± standard error and statistical comparisons were performed using one-way analysis of variance followed by the Tukey-Kramer HSD test for pair-wise comparisons. Pearson Chi-square test was used to analyze clinical features and TROP2 expression based on the calculated odds ratios (ORs) and 95% confidence interval (CI). Survival was evaluated using Kaplan-Meier curves and the difference was analyzed using the log-rank test. *P* < 0.05 was considered statistically significant.

## Results

### Tissue morphology and TROP2 expression across the clinical stages of OSCC

**Fig. 1:**
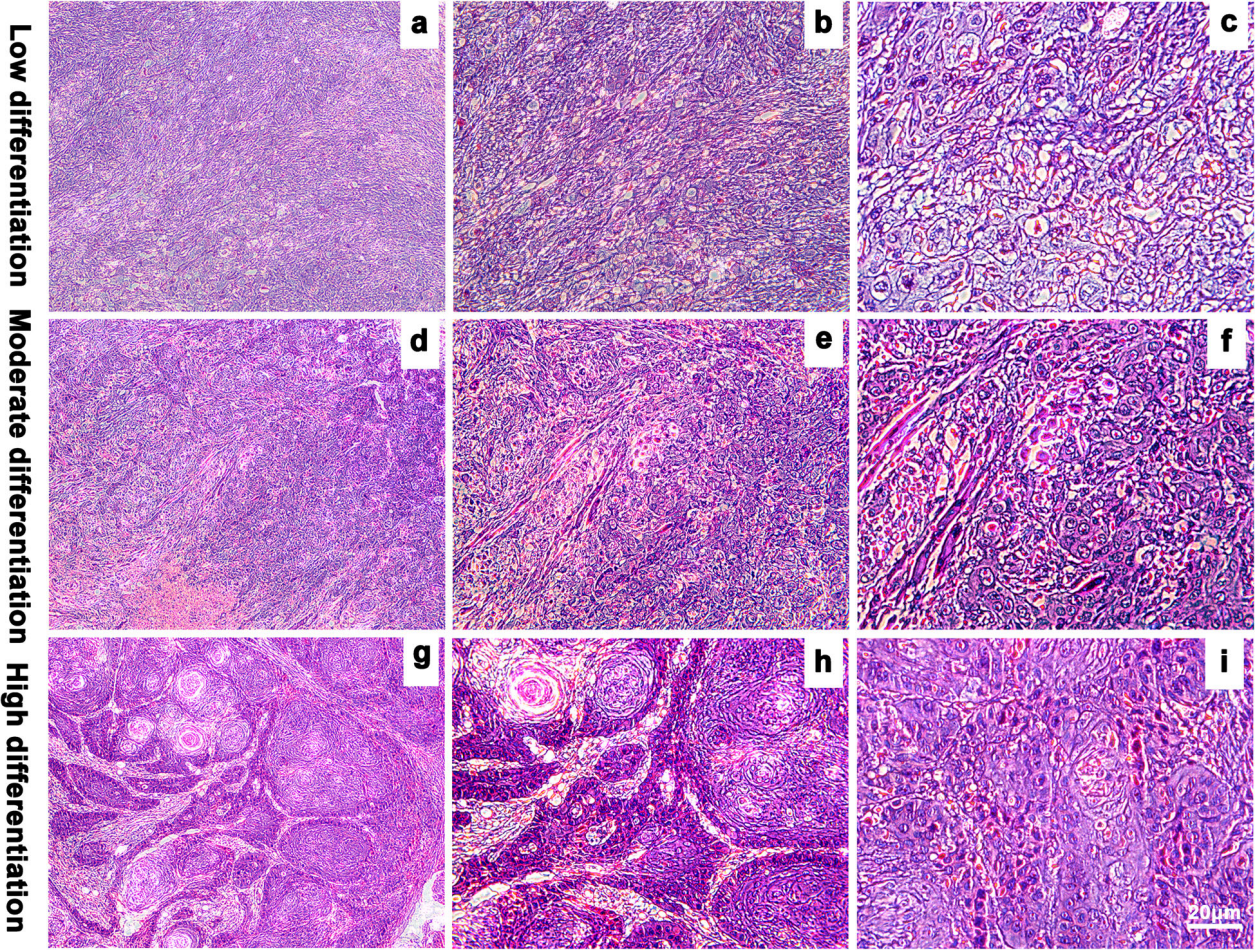
Paraffin pathological sections of tissues (a, d, g, × 4-fold; b, e, h, × 10-fold; c, f, i, × 40-fold)

**Fig. 2:**
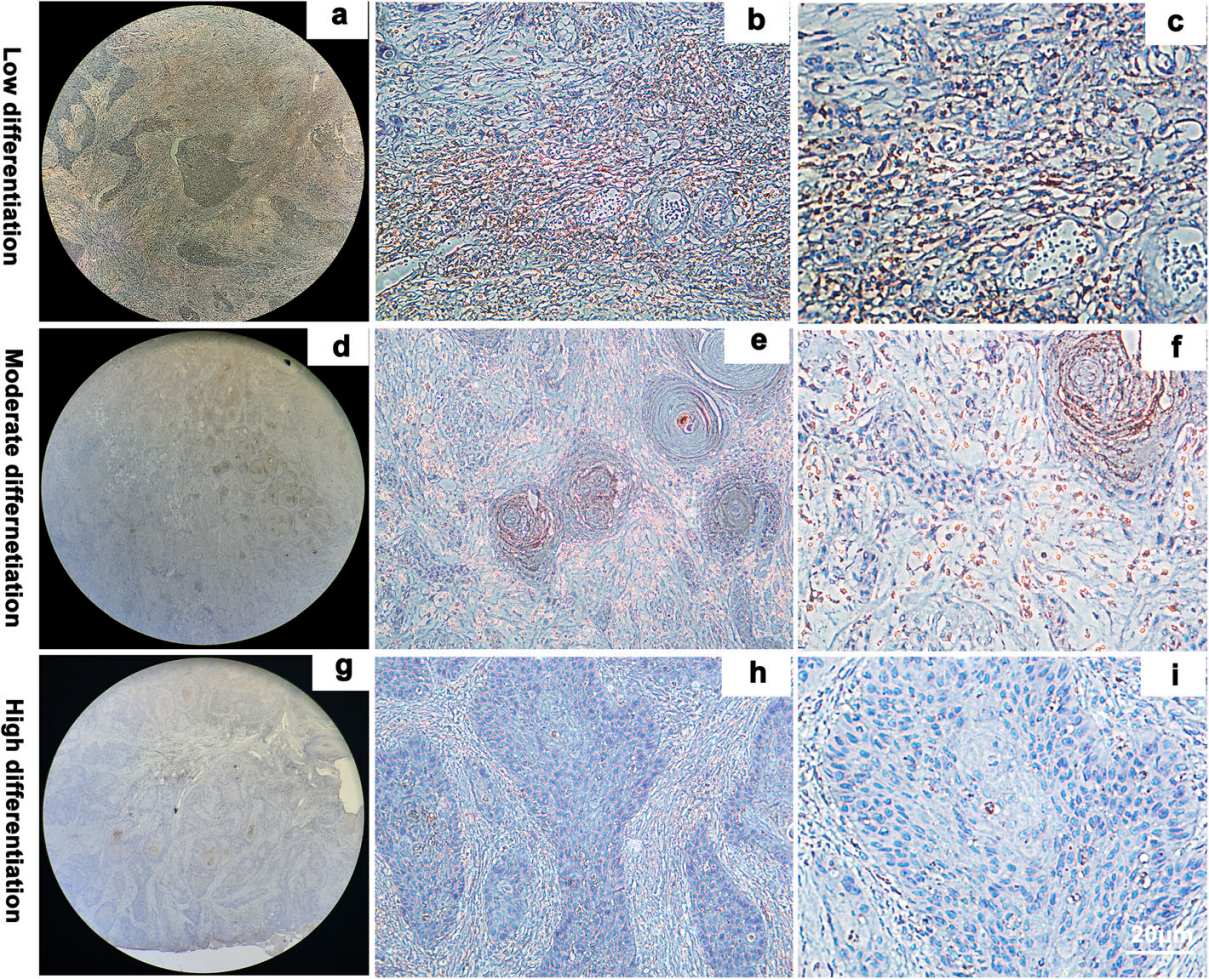
Immunohistochemical staining was performed to detect the expression of TROP2 at different stages of OSCC

**Fig. 3:**
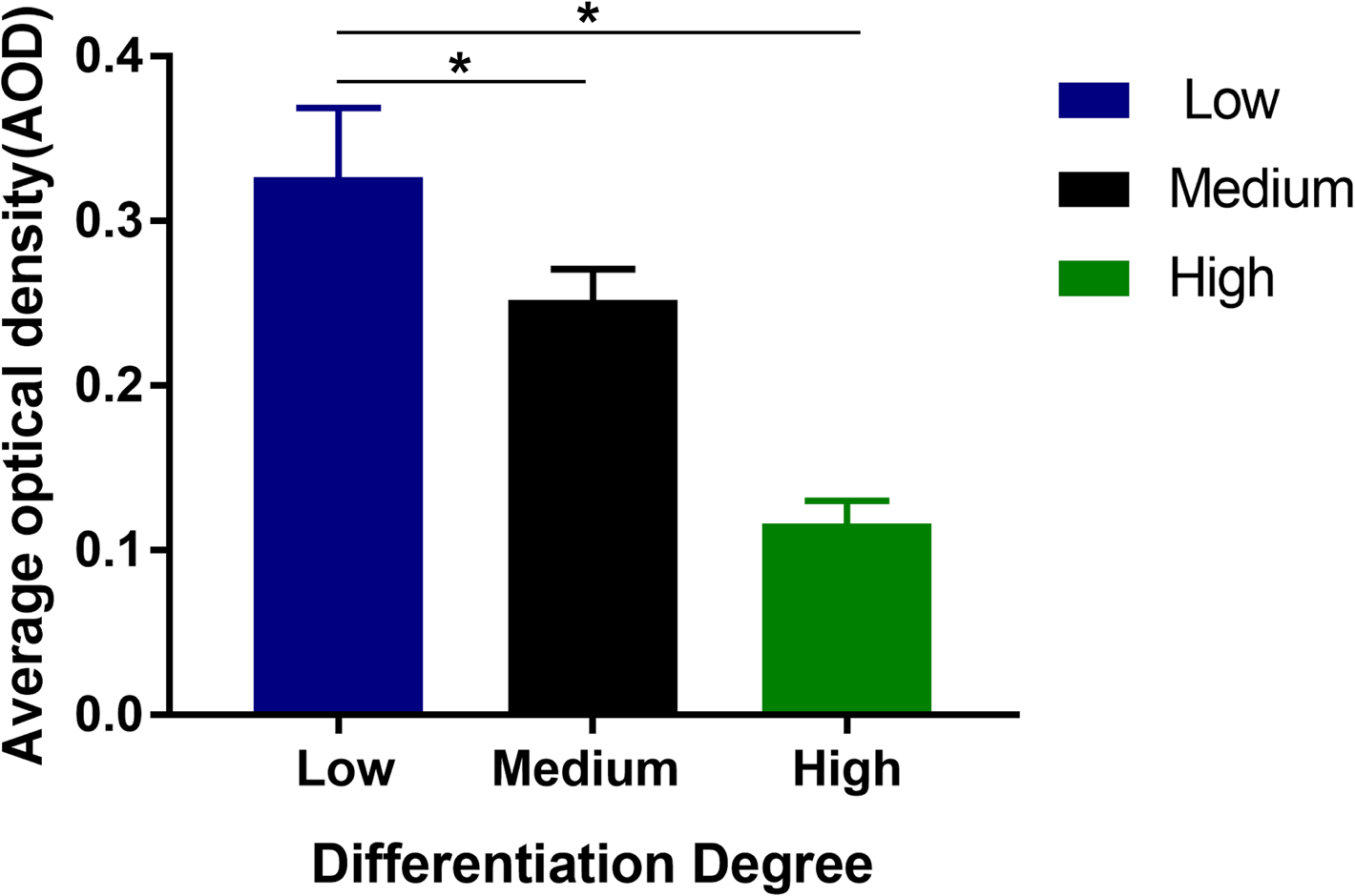
Average optical density of TROP2, poorly differentiated squamous cell carcinoma showed high expression(*P* < 0.05)

Tumor cells from poorly differentiated OSCC samples exhibited characteristic atypia, poor differentiation, and irregular morphology (Fig. 1). However, the number, volume, atypia, nuclear pyknosis, and mitotic structures decreased in tumor cells from highly differentiated OSCC as compared to those in poorly differentiated cells. TROP2 primarily localized in the cytoplasm of tumor cells, but not in adjacent normal epithelial cells. We observed that low differentiation and high malignancy of OSCC was associated with higher TROP2 expression (Fig. 2). The average optical density of TROP2 among the low, medium, and highly differentiated OSCC tissues were 0.32 ± 0.042, 0.25 ± 0.018, and 0.11 ± 0.013, respectively (Fig. 3).

### Association between TROP2 expression and clinical characteristics of OSCC

We analyzed the clinicopathological characteristics of patients with OSCC with varying degree of differentiation. Differential expression of TROP2 was associated with patient age, tumor differentiation, tumor size, TNM stage, percutaneous nerve filtration, and vascular invasion (Table 1, *P* < 0.05). Patients with poorly differentiated tumors were more likely than patients with well and moderate differentiated tumors to have high TROP2 expression (*P* < 0.001). However, there was no association between the expression of TROP2 and patient gender, tumor location, lymph node metastasis, or distant metastases (*P* > 0.05).

**Table 1:** Correlation between TROP2 expression and clinicopathological characteristics

Characters	n	TROP2 expression(%)		Pearson *x*^2^	*P* value
		Low or no(%)	High(%)		
Total	108	67	41		
Gender				0.786	0.375
Male	60	35	25		
Female	48	32	16		
Age				4.254	0.039
≥ 50	61	43	18		
<50	47	24	23		
Localization				2.217	0.136
mucosa	21	16	5		
Tongue	87	51	36		
Differentiation				77.268	< 0.001
well	36	35	1		
Moderate	36	31	5		
Poor	36	2	34		
Tumor size				32.883	< 0.001
T1 ≤ 2 cm	59	51	8		
2 cm<T2 ≤ 4 cm	49	16	33		
T3>4cm	0	0	0		
T4b	0	0	0		
Lymph node metastases				2.574	0.109
N0	42	30	12		
N_X_	66	37	29		
Distant metastases				1.015	0.314
M0	70	41	29		
M1	38	26	12		
TNM stage				67.880	< 0.001
I + II	78	67	11		
III + IV	30	0	30		
Perineural infiltration				16.881	< 0.001
No	91	64	27		
Yes	17	3	14		
Vascular invasion				27.688	< 0.001
No	45	41	4		
Yes	63	26	37		

### TROP2 expression and patient survival

**Fig. 4:**
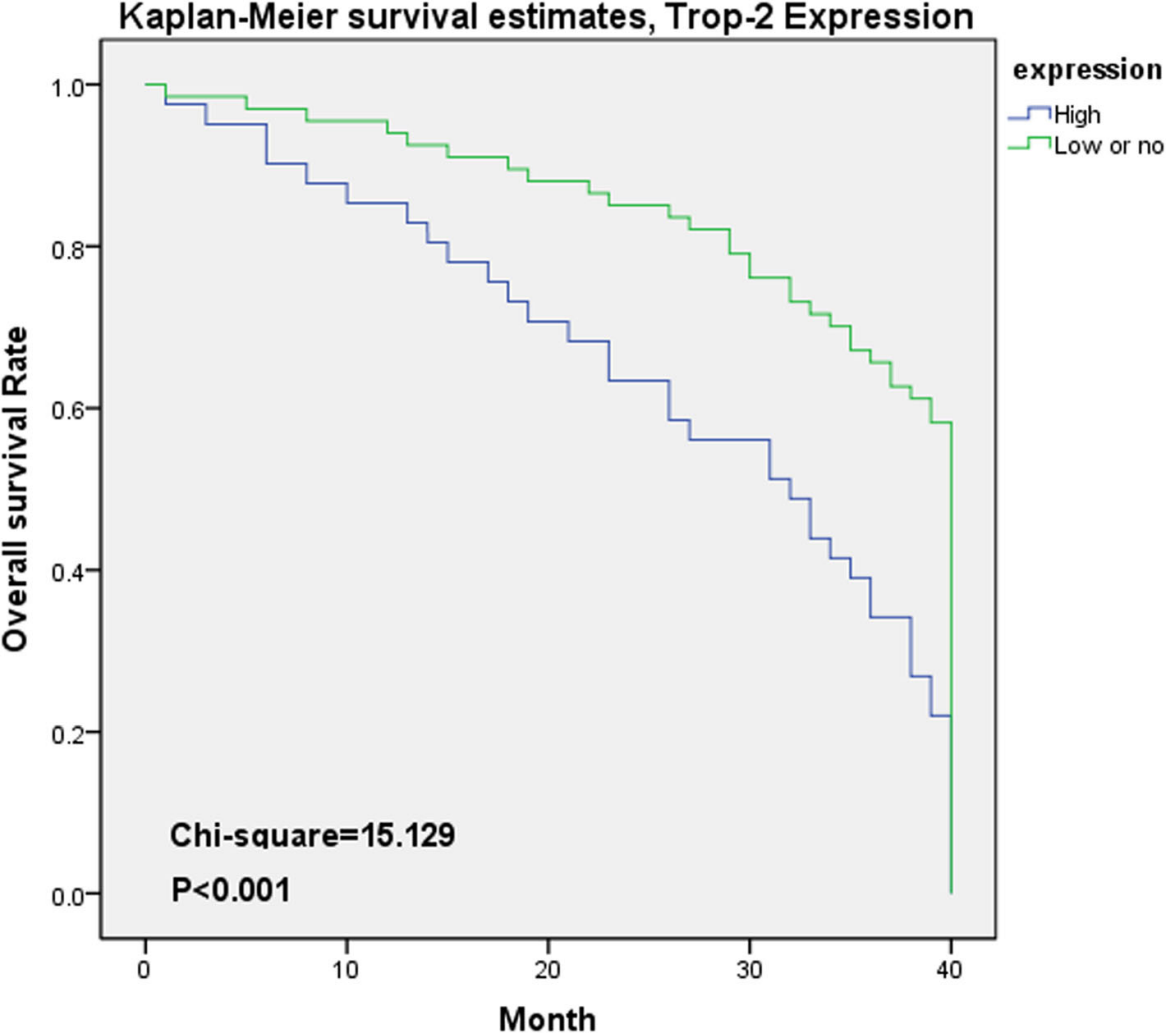
TROP2 total survival curve using Kaplan-Meier survival curves (low blue line, high green line)

Using Kaplan-Meier survival curves, we observed that an increase in TROP2 expression negatively correlated with the overall survival of patients (Fig. 4). And low/no of TROP2 expression group’s 3-years survival rate was 55.6%, a 20.4% for high expression group and 5-years rate were 45.4 and 10.2% respectively. TROP2 expression was associated with patient age (*P* = 0.039, OR = 0.437, 95% CI [0.198–0.966]), tumor differentiation (Well vs. Moderate, *P* > 0.05, OR = 5.645, 95% CI [0.625–50.987]; Moderate vs. Poor, *P* < 0.001, OR = 105.400, 95% CI [19.053–583.063]; Well vs. Poor, *P* < 0.001, OR = 595.000, 95% CI [51.529–6870.366]), tumor size (*P* < 0.05, OR = 13.148, 95% CI [5.060–34.168]), TNM stage (*P* < 0.05, OR = 0.141, 95% CI [0.082–0.244]), vascular invasion (*P* < 0.05, OR = 14.587, 95% CI [4.653–45.729]), and peripheral nerve invasion (*P* < 0.05, OR = 11.062, Table 2). High TROP2 expression was detected in older patients with low degree of differentiation, larger tumor volume, higher TNM staging, and vascular and peripheral nerve invasion, thereby resulting in lower overall survival. Thus, TROP2 may be a prognostic indicator for survival in OSCC patients.

**Table 2:** TROP2 expression risk factors with clinicopathological features

Characteristics	n	TROP2 expression(%)		*P* value	OR (95% CI)
		Low or no(%)	High(%)		
Total	108	67	41		
Gender				0.375	1.429 (0.649,3.147)
Male	60	35	25		
Female	48	32	16		
Age				0.039	0.437 (0.198,0.966)
≥ 50	61	43	18		
<50	47	24	23		
Localization				0.136	0.443 (0.149,1.318)
Mucosa	21	16	5		
Tongue	87	51	36		
Differentiation					
Well	36	35	1	> 0.05^a^	5.645 (0.625,50.987)
Moderate	36	31	5	< 0.001^b^	105.400 (19.053,583.063)
Poor	36	2	34	< 0.001^c^	595.000 (51.529,6870.366)
Tumor size				< 0.001	13.148 (5.060,34.168)
T1 ≤ 2 cm	59	51	8		
2 cm<T2 ≤ 4 cm	49	16	33		
T3>4cm	0	0	0		
T4b	0	0	0		
Lymph node metastases				0.109	1.959 (0.857,4.482)
N_0_	42	30	12		
N_X_	66	37	29		
Distant metastases				0.314	0.653 (0.284,1.501)
M0	70	41	29		
M1	38	26	12		
TNM stage				< 0.001	0.141 (0.082,0.244)
I + II	78	67	11		
III + IV	30	0	30		
Perineural infiltration				< 0.001	11.062 (2.939,41.641)
No	91	64	27		
Yes	17	3	14		
Vscular invasion				< 0.001	14.587 (4.653,45.729)
No	45	41	4		
Yes	63	26	37		

Note: ^a^, Well vs Moderate, ^b^, Moderate vs Poor, ^c,^ Well vs Poor

### Surface morphology and roughness of OSCC tissues

**Fig. 5:**
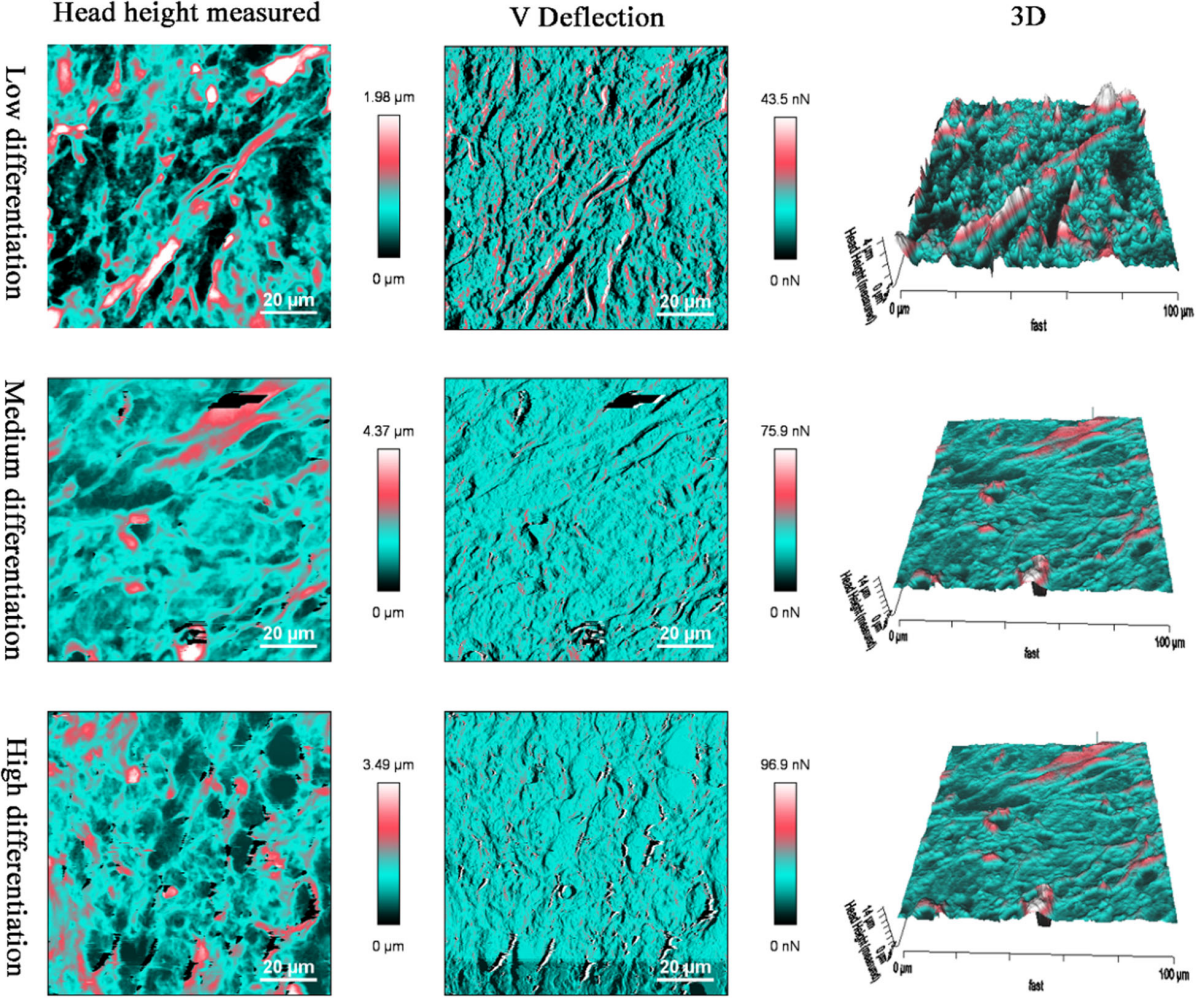
Surface morphology of OSCC tissue sections via AFM detection, irregular morphology appeared in the low differentiation

**Fig. 6:**
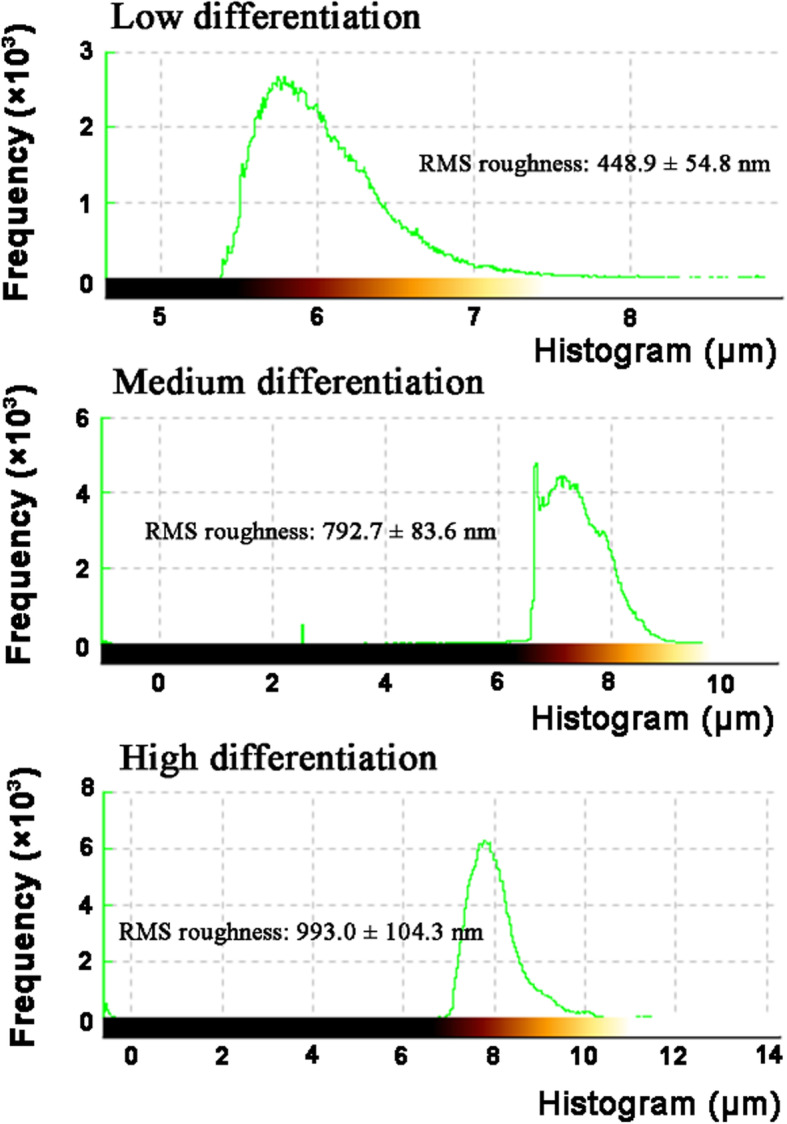
Surface roughness, results are express as mean ± SEM nm

The surface morphologies of OSCC tissues with varying degrees of differentiation were analyzed (direct topographical imaging) using BioAFM. Figure 5 shows the representative image from each tissue acquired during the cantilever-based AFM nano- indentation test. The tissue interface varied with tumor differentiation, indicating that highly differentiated OSCC tissues had a regular and flat morphology. OSCC tissues with low differentiation exhibited an overall irregular morphology with distinct modulation and loose tissue organization. Figure 6 summarizes the roughness of OSCC tissues with varying differentiation. The average surface roughness of low, medium, and highly differentiated OSCC tissues were 448.9 ± 54.8, 792.7 ± 83.6, and 993.0 ± 104.3 nm, respectively. Roughness of the tissue surface was enhanced with increasing differentiation of OSCC tissues.

### Young’s modulus of OSCC tissues

**Fig. 7:**
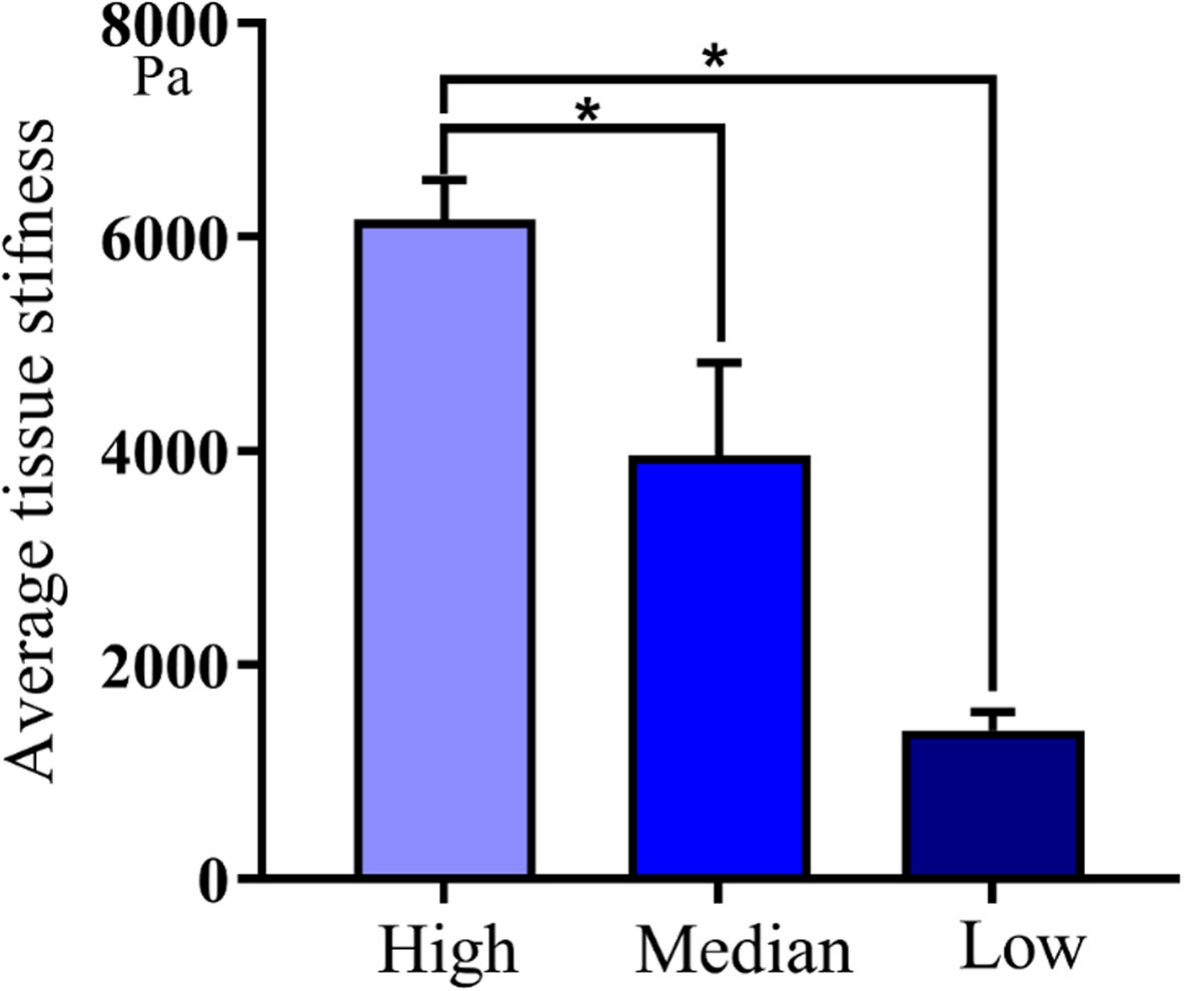
AFM test average tissue stiffness. Young’s modulus, E, was thus confirmed to be a parameter of cell hardness for various cells and tissue (Pa, *P* < 0.05)

We used BioAFM to determine Young’s modulus based on the mechanical properties of 108 OSCC tissues with varying degrees of differentiation. We randomly selected six contact points from each slice and each contact point was measured 15 times. Force-distance curves were generated for each slice and the JPK Data Processing software (5.1.8 version) was used to calculate Young’s modulus. Figure 7 shows the average variation in stiffness within individual tissues in the range of 1–8 kPa. In the low differentiation samples, we observed low stiffness as compared to that in high or medium differentiation samples (*P* < 0.05). Thus, tissue differentiation was positively associated with its stiffness (Fig. 7).

### Association between mechanical properties and TROP2 expression in OSCC

**Fig. 8:**
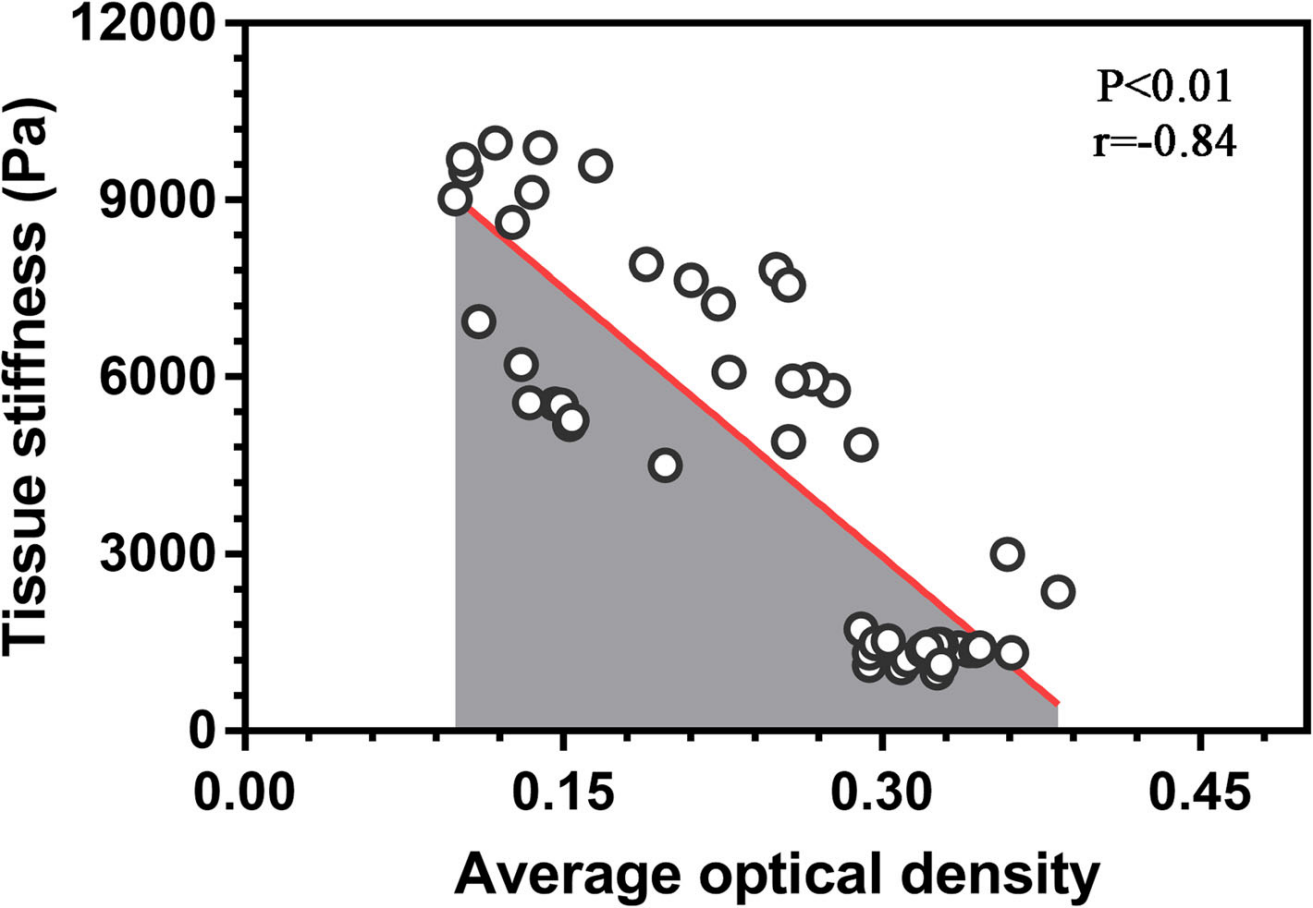
Correlation analysis between changes in mechanical stiffness of OSCC tissues and TROP2 expression Note: changes have statistical significance (*P* < 0.01) and show a certain negative correlation (*r* = − 0.84)

The Pearson coefficient showed a negative association between the stiffness of OSCC tissues and TROP2 expression (Fig. 8, *r* = − 0.84, *P* < 0.01). Thus, we detected an increase in stiffness with varying differentiation in the tumor samples.

## Discussion

TROP2 belongs to the family of genes involved in calcium signaling associated with tumorigenesis and found in human trophoblast and chorionic cell lines. Studies have shown that overexpression of TROP2 is associated with tumorigenesis and malignancy [28–30]. In this study, TROP2 expression was observed to be a highly sensitive and specific marker of tongue squamous cell carcinoma and tissue stiffness. The relative thickness of samples helped accurately diagnose and determine the staging of tongue squamous cell carcinoma.

Immunohistochemical analysis revealed that the expression of TROP2 in poorly differentiated OSCC tissues was significantly higher than that in well-differentiated OSCC tissues. Additionally, TROP2 upregulation was correlated with tumors of advanced TNM (III + IV) staging and poor differentiation than that in tumors with low TNM (I + II) staging. Thus, the abnormal expression of TROP2 may be associated with the occurrence and development of tongue malignancies. Furthermore, high TROP2 expression predicted low survival as compared to that in the tumors with low TROP2 expression. Previous research has also demonstrated the correlation between shorter patient lifespan and high levels of TROP2 as compared to that in patients with laryngeal squamous cell carcinoma and low levels of TROP2 [31]. TROP2 possesses sites for tyrosine/serine phosphorylation that regulate signal transduction or its expression and activity, thereby rendering cancer cells resistant to apoptosis [32]. Upregulated TROP2 correlates with the poor prognosis of thyroid papillary carcinoma [33], colon cancer [34], liver cancer [35], and other malignancies.

There have been an increasing number of studies on the biological role of TROP2 at the molecular level. TROP2 induces the downregulation/loss of PTEN, thereby stimulating PI3K/AKT signaling and tumor development [15]. PTEN is a well-known tumor suppressor that is a phosphatase [36] and affects the PI3K/PKB/AKT signaling axis during the dephosphorylation of PIP-2 and PIP-3 [37]. PI3K signaling is important in regulating tumor cell proliferation, migration, and invasion [38, 39]. Thus, PTEN is a negative regulator of cancer [40, 41]. Li et al. have shown that TROP2 activates epithelial-mesenchymal transition via PI3K/AKT signaling, thereby promoting proliferation, migration, and metastasis in gallbladder cancer [42]. Similarly, TROP2 expression stimulates the proliferation, migration, and invasion of osteosarcoma cells [43]. Hou et al. demonstrated that TROP2 regulates JAK2/STAT3 signaling in glioblastoma cells [44].

Functional differentiation of tissues influences the micro-morphology and mechanical stiffness of OSCC cells. We detected low surface roughness on OSCC tissues with loose structure, reduced hardness, and enhanced cell adhesion, migration, and invasion. Poorly differentiated OSCC tissues are “softer” than highly differentiated OSCC tissues. PI3K is an important cell-adhesion molecule. TROP2 triggers the synthesis of proteins with homologous domains, such as pleckstrin, RAC, Tiam, and Vav. Tiam and Vav activate RAC that leads to reorganization of the actin cytoskeleton, cell recognition, and adhesion [45].

The underlying mechanisms involved in the alteration of micromechanical properties of OSCC samples and occurrence, development, metastasis, and invasion of OSCC tumors remain to be elucidated. H&E staining is the gold standard for tumor diagnosis. With the development of biomechanics in the past two decades [46, 47], the mechanical properties of tissues need to be investigated based on biomedical and physical parameters. In this study, we have assayed the changes in mechanical properties at the micro-nanometer level using AFM and determined the association between the TNM grade, metastasis, and stiffness of tumor samples.

In conclusion, we have demonstrated the association between differential expression of TROP2 and patient age, tumor differentiation, tumor size, TNM stage, percutaneous nerve filtration, and vascular invasion. Moreover, high levels of TROP2 correlated with poor overall survival in patients. Highly differentiated cancer tissues exhibited increased surface roughness and stiffness. Lastly, high TROP2 expression resulted in reduced tumor stiffness. However, this study had some limitations. First, the cohort used in this study was relatively small. Second, we did not employ molecular methods of analysis such as western blotting or enzyme-linked immunosorbent assay. Thus, using a larger patient cohort and multiple techniques in molecular and cell biology will help validate our findings and develop TROP2 as a specific and efficient prognostic biomarker for OSCC.

## Conclusion

These findings could promote new methods for the early OSCC diagnosis depend on the stage of cancer and developing screening methods with high sensitivity and specificity. More detailed studies are needed to determine the feasibility and therapeutic benefit of testing tissue stiffness in human disease.

## Data Availability

The datasets used and analyzed during the current study are available from the corresponding author on reasonable request.

## Abbreviations

OSCC: Oral squamous cell carcinoma
TROP2: Trophoblast cell surface antigen 2
AFM: Atomic force microscopy
